# Gastric Cyto-Diagnosis

**Published:** 1914-12

**Authors:** 


**Gastric Cyto-Diagnosis,** Loeper and Binet, Arch. des Mal. de l'Appareil Digestif et de la Nutrition, have made 160 examinations, 21 followed to necropsy. After 12-hour fast, the stomach is washed, then 300 c.c. of 7:1000 NaCl solution is introduced and siphoned off after a minute, centrifuged for 10 minutes and the sediment smeared, dried, fixed with alcohol and stained with eosin-haematoxylin.

1. Normal—A few pavement cells of the mouth or esophagus, large, rectangular or polygonal, with small, dark nuclei; a small amount of debris. In cases of sialophagy these large cells with small nuclei are very numerous; they are usually covered with bacteria.

2. Dyspepsia—The same as above. Cystology here most useful in ruling out an organic lesion.

3. Gastritis—Pyotologically, four types are recognized:

(a) Mumous Gastritis—Characterized by an abundance of mucous; frequently yeast cells.

(b) Hypergenetic and Desquamative Gastritis—Very numerous, small, rounded polygonal cells; protoplasm is indistinct, granular, pale eosin color, and frequently escapes from the cell borders. Nuclei are relatively large and deeply stained blue. Differentiation between chief and border cells is impossible.

(c) Diapedetic Gastritis—Characterized by the large number of leucocytes, polymorphonuclear, eosinophilic and lymphocytic.

(d) Congestive Gastritis—Large number of red blood cells are present, besides the leucocytes. The red blood cells are dispersed, and pale, in contrast to the findings in ulcer, in which rouleaux formation and fresh, bright blood cells are seen.

In general the findings of the authors tend to increase the number of cases of gastritis at the expense of the dyspepsia cases.

4. Ulcer—A few small epithelial cells, many polymorphonuclear cells, and a variable number of red blood cells. Infected ulcers give a larger percentage of leucocytes. The process healing may be followed by the disappearance of this cystological formula.

5. Cancer—Pyloric tumors give cylindrical and elongated cells; those of the fundus and borders, small round and polygonal types. These latter have an indistinct protoplasm, light though large nuclei. The whole cell is smaller in size than a leucocyte.

Cells from colloid carcinomata show vacuolated protoplasm taking strong eosin stains.

Infection of an ulcerating growth is easily identified.

